# Case Report: Complete AV block in two patients with a congenital absence of the right coronary artery: an unusual correlation

**DOI:** 10.3389/fcvm.2025.1556188

**Published:** 2025-03-07

**Authors:** Ruihui Lai, Shuai Sun, Yanjun Chen, Tan Xu

**Affiliations:** ^1^ Department of Cardiology, Peking University Shenzhen Hospital, Shenzhen, China; ^2^ Department of Cardiology, Shantou University Medical College, Shantou, China

**Keywords:** congenital absence of the right coronary artery, complete atrioventricular block, coronary angiography, arrhythmia, case report

## Abstract

**Background:**

Congenital absence of the right coronary artery (RCA) is an extremely rare coronary anomaly with a very low incidence in the general population. The prevalence of complete atrioventricular (AV) block also appears to be low. No previous reports have documented the coexistence of congenital absence of the RCA and complete AV block in the same patient.

**Case summaries:**

Case 1 was a 52-year-old man with no significant past medical history who experienced syncope. The initial ECG revealed complete AV block with a non-specific ST-T segment. Coronary angiography showed mild, non-obstructive atherosclerosis in the dominant left circumflex artery (LCx), which continued along the anatomical course of the RCA. The patient underwent a dual-chamber pacemaker implantation for complete AV block. Case 2 was a 79-year-old man with a history of hypertension and coronary heart disease who presented with gradually worsening fatigue lasting 6 h. ECG showed complete AV block with a non-specific ST-T segment. Coronary angiography revealed an abnormal origin of the RCA arising from the distal portion of a dominant LCx, which retrogradely followed the course of a normal RCA to the base of the heart. The patient also underwent a dual-chamber pacemaker implantation for complete AV block.

**Conclusion:**

These two cases represent the first reported instances of complete AV block coexisting with congenital absence of the RCA, where the LCx supplied the territory of the RCA without evidence of myocardial infarction.

## Graphical Abstract

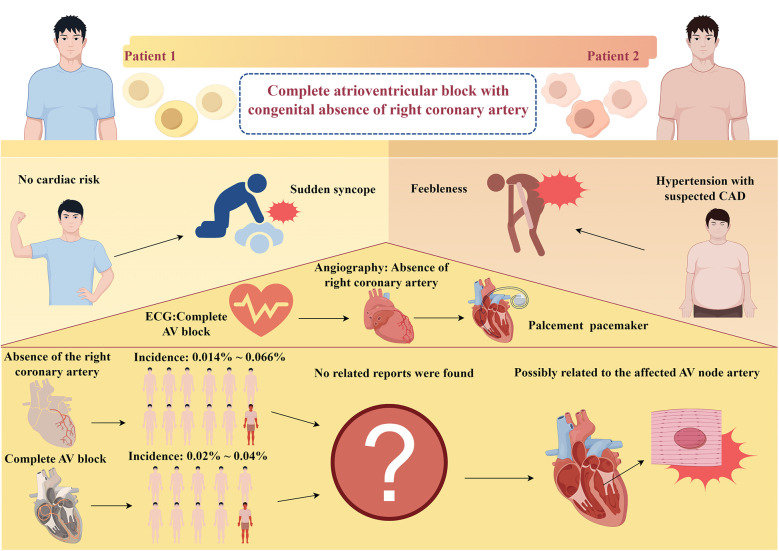

Two cases represent first reported instances of complete AV block coexisting with congenital absence of the RCA, where the LCx supplied the territory of the RCA without evidence of myocardial infarction.

## Highlights

• Rarity of congenital absence of right coronary artery: Congenital absence of the right coronary artery (RCA) is a rare form of coronary artery disease with an extremely low incidence in the general population, estimated to be approximately 0.014%–0.066%.
• Uncommon nature of complete AV block: Although atrioventricular (AV) block is relatively common, complete AV block is relatively rare with a prevalence of approximately 0.02%–0.04%.
• Novel coexistence of RCA absence and complete AV block: The two cases demonstrate the coexistence of complete AV block with an isolated single coronary artery and an absent RCA, where the LCx supplies the territory of the RCA without evidence of myocardial infarction.

## Introduction

Congenital single coronary artery (SCA) is a rare anomaly in which only one coronary artery originates from a single coronary ostium to supply the entire heart (1, 2). The incidence of an SCA ranges from 0.024% to 1% in various reports involving over 1.4 million patients (1, 3, 4). SCA is sometimes associated with congenital cardiac structural abnormalities, such as pulmonary artery atresia, tetralogy of Fallot, and patent truncus arteriosus (5). Congenital absence of the right coronary artery (RCA) is a form of SCA with an extremely low incidence in the general population, estimated to be approximately 0.014%–0.066% (6).

**Figure 1:**
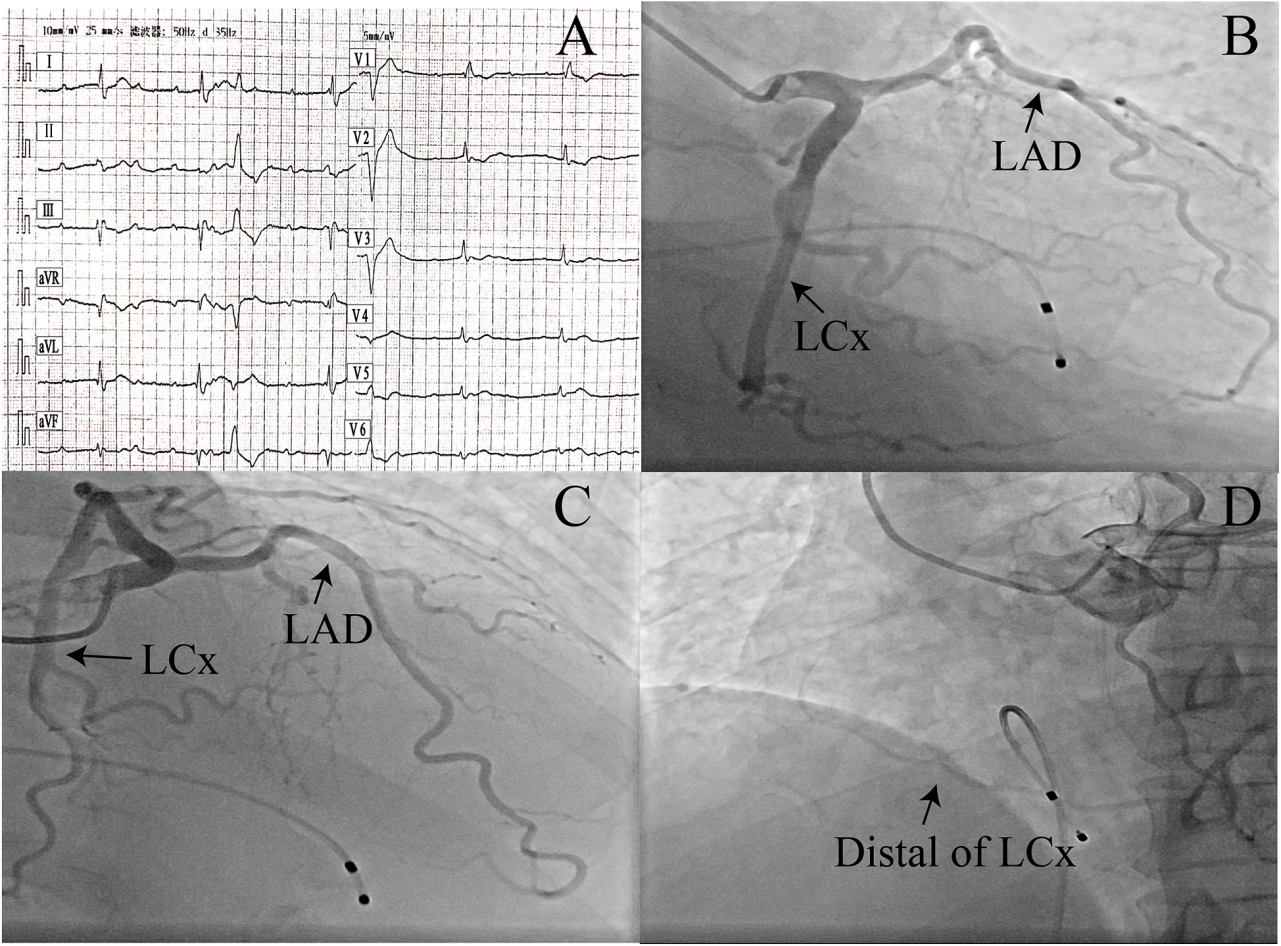
**(A)** Initial ECG in the emergency department: complete atrioventricular block and a non-specific ST-T segment. **(B–D)** Coronary angiography showing different coronary arteries and the left circumflex artery supplying the right coronary artery region (angiographic projections: **B**, RAO 26°/CAUD 30°; **C**, RAO 26°/CRAN 28°; **D**, LAO 38°/CAUD 3°). LAD, left anterior descending artery; LCx, left circumflex artery; RAO, right anterior oblique; CAUD, caudal; CRAN, cranial; LAO, left anterior oblique.

Although atrioventricular (AV) block is relatively common, complete AV block is relatively rare (7). The prevalence in the general population appears to be low, approximately 0.02%–0.04% (8). In apparently healthy and asymptomatic individuals, the incidence of complete AV block is as low as 0.001% (9).

To date, there have been no reports of the coexistence of congenital absence of the RCA and complete AV block without a reversible cause. We present two cases of complete AV block coexisting with congenital absence of the RCA without evidence of myocardial infarction.

## Summary figure

## Case presentation

### Case 1

A 52-year-old man with no past medical history or cardiac risk factors was brought to the emergency department following an episode of syncope. Initial ECG findings indicated complete AV block with a non-specific ST-T segment (Figure 1A). The patient’s cardiac troponin T was slightly elevated (0.030 ng/mL, normal reference range <0.014 ng/mL), while cardiac troponin I was within the normal range (0.026 ng/mL, normal reference range <0.034 ng/mL). His family history was negative and free of cardiac events. A physical examination revealed bradycardia but no other significant abnormalities. No edema or congestion signs were found. A transthoracic echocardiographic examination and chest x-ray showed no obvious abnormality. The patient experienced another episode of syncope due to a long RR interval and was immediately implanted with a temporary pacemaker. Suspecting acute myocardial infarction, the patient underwent coronary angiography (Figures 1B–D and Supplementary Videos 1–3). The angiogram revealed that the left anterior descending artery (LAD) and the left circumflex artery (LCx) originated from the left main coronary artery. Mild, non-obstructive atherosclerosis was observed in the dominant LCx, which continued along the anatomical course of the RCA. An injection into the right sinus of Valsalva unveiled the absence of a right coronary ostium separate from the aorta. When arriving at a diagnosis for this case, considerations of differential diagnoses, such as right coronary artery occlusion, were taken into account. No additional imaging tests, such as CT scanning or cardiac MRI, were performed, as the vessel course was clear on angiography alone. Five days later, the ECG still showed complete AV block with a low heart rate, and the patient then underwent a dual-chamber pacemaker implantation. At follow-up visits, the patient reported no discomfort.

**Figure 2:**
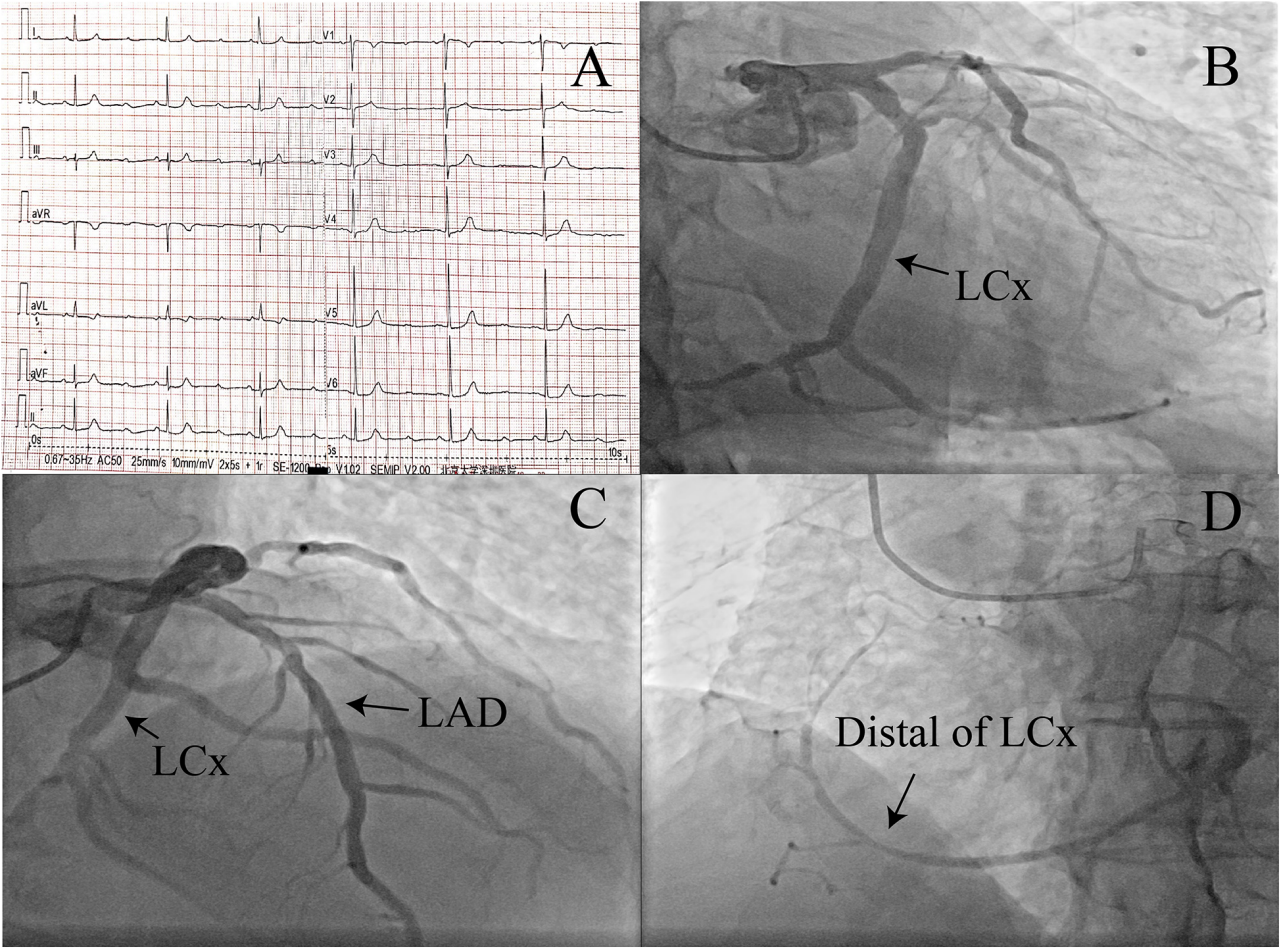
**(A)** ECG in the emergency department: complete atrioventricular block and a non-specific ST-T segment. **(B–D)** Coronary angiography showing different coronary arteries and the left circumflex artery supplying the right coronary artery region (angiographic projections: **B**, 0/CAUD 31°; **C**, RAO 2°/CRAN 36°; **D**, LAO 40°/CAUD 2°). LAD, left anterior descending artery; LCx, left circumflex artery; RAO, right anterior oblique; CAUD, caudal; CRAN, cranial; LAO, left anterior oblique.

**Figure 3:**
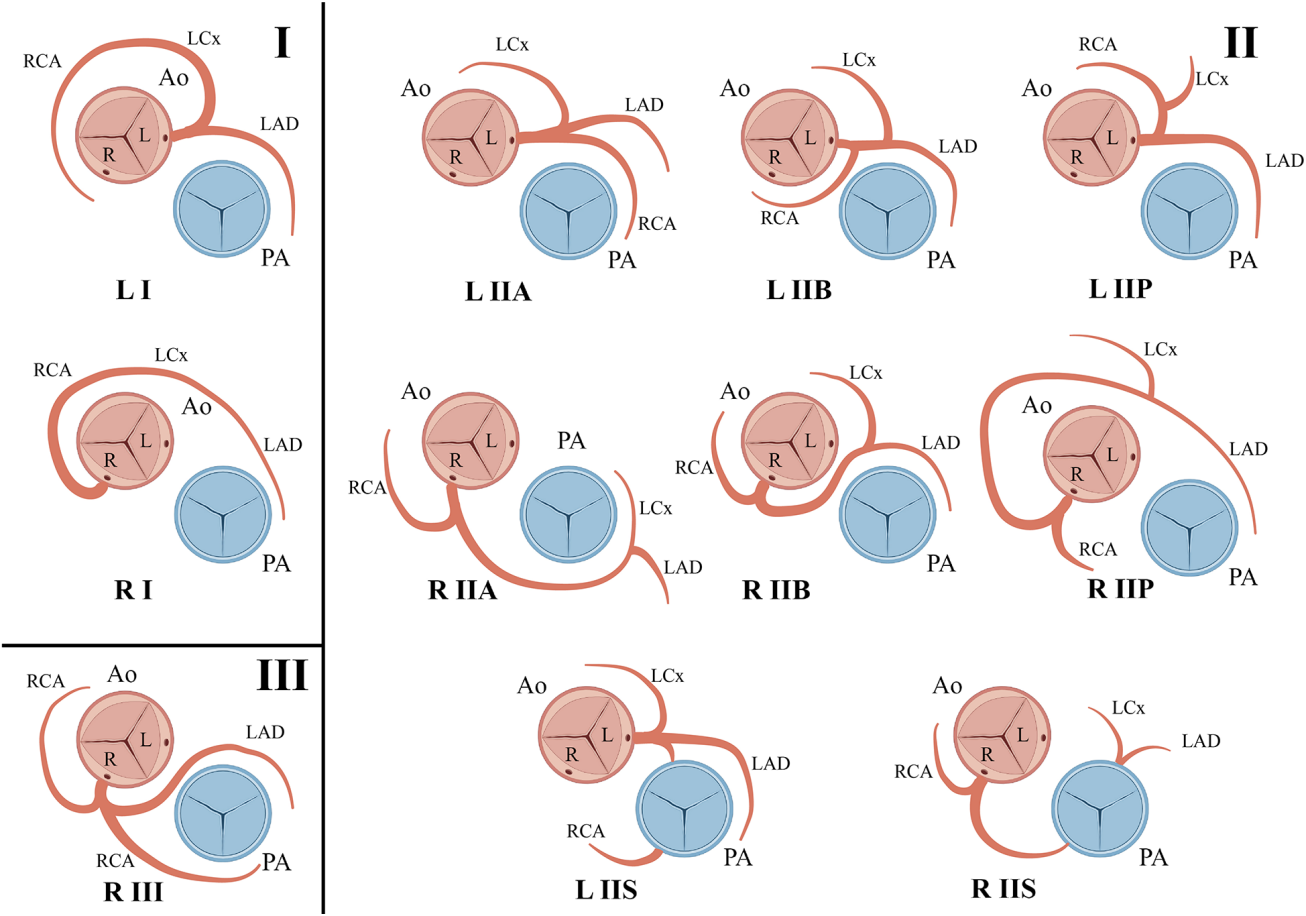
The letters R or L are used to identify the ostial origin of the vessel; the Roman numerals I, II, or III are used to represent the anatomical distribution of the vessel; and the letters A, B, P, S, and C are used to delineate the course of the vessel in relation to the pulmonary artery and the aorta.

### Case 2

A 79-year-old man with a history of hypertension and coronary heart disease presented to his general practitioner clinic with gradually worsening fatigue lasting 6 h. His heart rate was 30 beats per min (bpm), and his blood pressure was 170/100 mmHg. The patient was immediately transported to the emergency room.

On admission, his vital signs showed a heart rate of 45 bpm, blood pressure of 104/42 mmHg, and a temperature of 36.5 °C. The physical examination was otherwise unremarkable. ECG showed complete AV block with a non-specific ST-T segment and a heart rate of 38 beats/min (Figure 2A). Laboratory findings, which included cardiac troponin, were within normal limits. A transthoracic echocardiographic examination and chest x-ray showed no obvious abnormalities.

Given the history of suspected coronary heart disease, the patient was scheduled for coronary angiography (Figures 2B–D and Supplementary Videos 4–6). During catheterization, only one coronary ostium originating from the left coronary cusp could be cannulated, and several attempts with different catheters to identify the RCA ostium failed. The patient had an SCA arising from the left coronary cusp. The RCA had an abnormal origin from the distal end of a dominant LCx that retrogradely followed the course of a normal RCA to the base of the heart. The flow of the LCx and LAD was normal, with mild stenosis. Similar to the first patient, the ECG still showed complete AV block with a low heart rate after 5 days. The patient also underwent a dual-chamber pacemaker implantation. At follow-up visits, the patient reported no discomfort.

## Discussion

We are the first to report two rare cases of complete AV block coexisting with isolated SCAs and absent RCAs, in which the distal portion of the LCx supplied the territory of the RCA.

In 1979, Lipton et al. proposed the angiographic classification of SCA (6), which was later modified by Yamanaka and Hobbs (4). SCAs can be classified into three groups (Figure 3). This classification takes into account variables such as the origin of the ostium from the sinus of Valsalva, the anatomical course of the vessel, and the course of the transverse trunk. According to this system, our patients would be classified as LI. Many patients with an SCA are asymptomatic at the time of diagnosis, and cases of SCA are often discovered incidentally during coronary angiography, as in our patients (2). The majority of patients may experience atypical chest pain or non-specific symptoms in the absence of obstructive coronary artery disease (10). Others may present with typical chest pain; sudden death, especially during exercise; syncope; palpitations; ventricular tachycardia; or myocardial infarction (11). Certain anomaly classifications, such as RI and LI, typically have a benign clinical course.

The coexistence of complete AV block and coronary disease is primarily associated with acute myocardial infarction (12). The majority of cases of complete AV block are transient following revascularization of the culprit artery (13). Patients with congenital absence of the RCA may also experience acute myocardial infarction with complete AV block (14). However, there are no previous reports of complete AV block coexisting with congenital absence of the RCA in the absence of myocardial infarction. This may be due to the extremely low incidence of both SCA anomalies and complete AV block in the general population.

Degeneration and compromised blood supply to the AV nodal artery may be the most likely causes of complete AV block in the two cases. A previous case report showed an association between the coronary slow flow phenomenon and AV block (15). In that case, the patient underwent coronary angiography, which revealed a coronary slow flow phenomenon without significant stenosis. Due to persistent AV block, the patient was discharged following permanent pacemaker implantation (15). In 2018, the “FIT Clinical Decision Making” program published in the *Journal of the American College of Cardiology* reported a case of complete AV block with an anomalous RCA (16). Coronary computed tomography angiography revealed an anomalous origin of the RCA from the left cusp and a course between the aorta and the pulmonary artery (16). An electrophysiology study revealed multilevel intrahisian block and the patient underwent a dual-chamber pacemaker implantation.

## Conclusion

Here, we reported the first two cases of congenital RCA absence with complete AV block in the absence of myocardial infarction. The coexistence of these two rare entities underscores the importance of anatomical and functional coronary evaluation in atypical arrhythmia presentations, expanding our understanding of the non-ischemic causes of complete AV block.

## Data Availability

The original contributions presented in the study are included in the article/Supplementary Material, further inquiries can be directed to the corresponding author.
